# Intraoperative Allogeneic Blood Transfusion Has No Impact on Postoperative Short-Term Outcomes After Pancreatoduodenectomy for Periampullary Malignancies: A Propensity Score Matching Analysis and Mediation Analysis

**DOI:** 10.3390/cancers16203531

**Published:** 2024-10-18

**Authors:** Kristjan Ukegjini, René Warschkow, Henrik Petrowsky, Philip C. Müller, José Oberholzer, Ignazio Tarantino, Jan Philipp Jonas, Bruno M. Schmied, Thomas Steffen

**Affiliations:** 1 Department of Surgery, Kantonsspital St. Gallen, 9007 St. Gallen, Switzerland; rene.warschkow@kssg.ch (R.W.); ignazio.tarantino@kssg.ch (I.T.); bruno.schmied@kssg.ch (B.M.S.); thomas.steffen@kssg.ch (T.S.); 2 Department of Surgery and Transplantation, Swiss HPB & Transplant Center Zurich, University Hospital Zurich, 8091 Zürich, Switzerland; henrik.petrowsky@usz.ch (H.P.); jose.oberholzer@usz.ch (J.O.); janphilipp.jonas@usz.ch (J.P.J.); 3 Department of Surgery, Clarunis—University Centre for Gastrointestinal and Hepatopancreatobiliary Diseases, 4031 Basel, Switzerland; philip.mueller@clarunis.ch

**Keywords:** intraoperative allogeneic blood transfusion, short-term outcomes, pancreatoduodenectomy

## Abstract

**Simple Summary:**

The effect of a perioperative blood transfusion on the postoperative short-term outcomes of patients with periampullary malignancies following pancreatoduodenectomy is still a topic of debate within the literature. To address this issue, a partial correlation and mediation analysis were integrated with propensity score matching and weighting, with the objective of examining the influence of intraoperative blood transfusions on postoperative outcomes. After adjusting for intraoperative blood loss, intraoperative transfusion was not found to be associated with postoperative complications in patients undergoing pancreatoduodenectomy. From a clinical perspective, this finding has important implications, as it allows for the consideration of transfusions in pancreatoduodenectomy patients on an as-needed basis, with the confidence that this will not adversely impact postoperative outcomes.

**Abstract:**

**Background/Objectives**: The aim of this study was to investigate the association between intraoperative blood transfusion (BT) and the short-term outcomes of pancreatoduodenectomy (PD) for patients with periampullary malignancies. **Methods**: In a retrospective two-center cohort analysis, we utilized a logistic and mixed-effects ordinal regression, nonparametric partial correlation, and mediation analysis, complemented by propensity score matching (PSM) and weighting. **Results**: A total of 491 patients were included. Of these, 18 (3.7%) received an intraoperative BT. An intraoperative BT was associated with blood loss (odds ratio (OR) per 100 mL = 1.42; 95% CI 1.27 to 1.62; *p* < 0.001) and relatively high ASA classes (OR = 3.75; 95% CI 1.05 to 17.74; *p* = 0.041). Intraoperative blood loss (r = 0.27; *p* < 0.001) but not intraoperative BT (r = 0.015; *p* = 0.698) was associated with postoperative complications. Intraoperative BT was associated with postoperative complications according to the unadjusted regression (OR = 1.95; 95% CI 1.38–2.42, *p* < 0.001) but not the multivariable ordinal regression. In the mediation analysis for relative risk (RR), intraoperative BT was beneficial (RR = 0.51; 95% CI: 0.01–0.78), and blood loss (RR = 2.49; 95% CI: 1.75–177.34) contributed to the occurrence of major postoperative complications. After PSM, analyses revealed that an intraoperative BT did not have a significant impact on the rates of postoperative major complications (OR = 1.048; *p* = 0.919), clinically relevant postoperative pancreatic fistula (OR = 0.573; *p* = 0.439) or postoperative 90-day mortality (OR = 0.714; *p* = 0.439). **Conclusions**: When adjusting for intraoperative blood loss, intraoperative BT is not associated with postoperative complications.

## 1. Introduction

Pancreatoduodenectomy (PD) with regional lymph node dissection represents the only curative treatment for periampullary malignancies. Patients presenting with periampullary carcinoma have an increased risk of perioperative anemia, which necessitates the administration of a perioperative allogeneic blood transfusion (BT). This outcome results from blood loss due to the underlying disease and bone marrow suppression resulting from neoadjuvant treatments [1]. Furthermore, the performance of PD can result in substantial intraoperative blood loss, so a perioperative BT may be required [2].

The impact of a perioperative BT on the postoperative short-term outcomes and prognosis of patients with periampullary malignancies remains a topic of ongoing debate. Previous studies have indicated that a perioperative BT can increase the risks for perioperative morbidity [3,4,5] and cancer recurrence despite good disease-free survival and overall survival [5,6,7,8,9,10]. However, in some studies, researchers have not reported a negative impact of perioperative BT on postoperative outcomes. Further studies are needed to determine the postoperative short-term outcomes of BT in patients with periampullary malignancies and the relationship between BTs and the type of PD and estimated blood loss volume.

As previously stated, some studies have compared perioperative BT with postoperative short-term complications. It should be noted that the term ‘perioperative BT’ encompasses a heterogeneous cohort comprising both patient groups who received intraoperative BT and those who received postoperative BT. The difficulty in establishing an association between postoperative BT and postoperative complications such as postoperative pancreatic fistula (POPF) and postpancreatectomy hemorrhage (PPH) is due to the fact that postoperative BTs are initiated following the occurrence of these events. As a result, it is challenging to discern the relationship between postoperative complications and postoperative BT. Consequently, a study of intraoperative BT is suitable for verifying the relationship in question. To our knowledge, only one study from 2002 [11] has examined the prognostic significance of intraoperative BT after PD. No study has examined the relationship between intraoperative BT and postoperative short-term outcomes in patients who have undergone PD.

The aim of this study was to assess the putative effect of intraoperative BT on postoperative short-term outcomes in patients undergoing PD, utilizing recent statistical methods to minimize potential bias.

## 2. Materials and Methods

### 2.1. Study Design

This retrospective two-center cohort study was conducted following the Strengthening the Reporting of Observation in Epidemiology (STROBE) guidelines [12] and the principles of the Declaration of Helsinki. The respective ethical committees of the Canton of St. Gallen and the Canton of Zurich approved a formal ethical review. (BASEC Nr.: 2024-00827).

All patients who underwent PD between December 2008 and March 2022 at the Department of Visceral and Transplant Surgery at the University Hospital of Zurich and the Department of General, Visceral, Endocrine, and Transplant Surgery at the Kantonsspital St. Gallen were screened for inclusion in this study. Data relevant to this study were gathered from two prospectively maintained databases.

### 2.2. Inclusion and Exclusion Criteria

Patients were included if they met the following criteria: 18 years old or older, had undergone PD for histologically proven periampullary malignancies, had undergone intraoperative BT, and were followed up for at least 30 days.

To ensure homogeneity, patients with incomplete data and patients for whom no data on intraoperative BTs, blood loss, or hemoglobin (Hb) values were available were excluded from the analysis.

### 2.3. Intraoperative Patient Blood Management

In accordance with the World Health Organization, anemia is defined as a Hb level below 130 g/L for males and 120 g/L for females. A preoperative screening process was implemented to identify any patients exhibiting indications of anemia. In the event of a diagnosis of anemia, the commencement of iron replacement therapy was considered, in accordance with feasibility, as an appropriate course of action to address the underlying anemia.

Intraoperative BT was defined as the transfusion of allogeneic packed erythrocytes during surgery. The packed blood cells were stored in a citrate-phosphate-dextrose-adenine anticoagulant solution devoid of leukodepletion. In general, the indication for intraoperative BT was an Hb concentration of less than 70 g/L prior to the commencement of PD. The potential for active bleeding should be considered during this procedure [13]. However, BT was also permitted in cases of active bleeding during PD, which led to a reduction in the Hb value. Patients without a history of coronary heart disease received a transfusion only if the Hb level fell below 70 g/L. Patients with coronary heart disease or clinical indications received a transfusion only if the Hb level fell below 80 g/L.

### 2.4. Definition of Outcomes

The primary outcome of the study was the incidence of postoperative complications following PD in patients with and without intraoperative blood transfusion. Postoperative complications were graded according to the Clavien-Dindo (CD) classification [14], with major complications defined as CD grade III or higher complications. In order to evaluate the relationship between intraoperative BT and postoperative complications with respect to CD, a number of potential sources of confounding, including intraoperative blood loss, the duration of surgery, and the rate of PD severity, were incorporated into multivariable statistical models.

The secondary outcomes included the incidence of pancreas-specific morbidities, surgical site infection (SSI), reoperation rate, total length of intensive care unit (ICU) stay, postoperative length of hospital stay (LOS), and mortality rates at 30 days and 90 days. Pancreas-specific morbidities, such as POPF [15], PPH [16] and delayed gastric emptying (DGE) [17], were classified according to the International Study Group of Pancreatic Surgery (ISGPS). Bile leakage was classified according to the International Study Group of Liver Surgery (ISGLS) [18]. The SSIs were defined in accordance with the Centers for Disease Control and Prevention (CDC) criteria [19,20]. The types of PD were classified according to Mihaljevic et al. [2] (PD Type I: standard PD; PD Type II: PD with portal vein/superior mesenteric vein resection; PD Type III: PD with multi-visceral resection; and PD Type IV: PD with arterial resection).

### 2.5. Follow-Up

Following discharge, the study nurses conducted regular telephone interviews with patients to ascertain their postoperative status. Patients who had been discharged with drains were observed for follow-up during regular office hours. Each patient was seen in the office at the 90-day follow-up.

### 2.6. Statistical Analysis

The statistical analysis was performed using R statistical software version “R 4.4.0” (www.r-project.org (accessed on 30 July 2024)). A two-sided *p* value less than 0.05 (2-sided) indicated statistical significance. Confidence intervals for binomial probabilities were estimated using the Agresti and Coull method. Categorical and continuous baseline characteristics were compared using chi-square tests and Mann-Whitney U tests, respectively. A multivariate logistic regression was performed to identify independent predictors of intraoperative BT. Categorical and continuous outcomes were compared using chi-square tests and Mann-Whitney U tests. The relationships among the number of intraoperative BTs, CD grade, blood loss volume, and preoperative hemoglobin level were analyzed via a nonparametric partial correlation analysis on the basis of the Spearman rank correlation coefficient. To further assess the effect of intraoperative BT on the Clavien–Dindo grade, a generalized ordinal mixed model with the main surgeon nested under the two study centers, PD difficulty and stage as random effects fitted with uninformative priors by the Markov chain Monte Carlo sampler using the MCMCglmm R package with 3000 iterations after 1000 burnins and a thinning factor of 1 was performed. Thereafter, a mediation analysis [21] was conducted using an inverse odds ratio-weighted (IORW) approach [22] to further elaborate the effects of intraoperative BT on blood loss volume and CD grade, with adjustments for preoperative Hb level, PD difficulty, duration of surgery, sex, and body mass index (BMI). The effects were deemed causal and estimated via direct counterfactual imputation estimation with bias-corrected and accelerated (BCa) bootstrap 95% CIs using the R library CMAverse [22].

To further minimize differences in the baseline characteristics between the intraoperative BT and non-intraoperative BT groups, propensity score matching (PSM) was performed using the R MatchIt library [23,24,25] because of the high likelihood of relevant bias introduced by BT. Full bipartite matching and weighting were utilized for matching one to many patients in one group with a patient in the other group. The balance between the two groups was further diminished by weight. PSM was estimated with logistic regression. After searching the literature, potentially influential confounding variables were determined (intraoperative blood loss, duration of surgery, PD severity, age, sex, BMI, and ASA class), and second-order interactions between the continuous confounders were used to estimate the propensity score. The caliper width was set to 0.5 to obtain an optimal balance. After PSM, the maximal standardized mean difference (SMD) was 0.07, indicating a good balance [26]. In each group, each patient was matched with a patient in the other group, and weights were assigned such that at the end of this procedure, there was a virtually similar propensity score in the two groups. There was no match for 373 patients in the non-intraoperative BT group or for 5 patients in the intraoperative BT group; therefore, these patients were excluded from the analysis. To control for balance and inference after PSM, the marginal average treatment effects of intraoperative BT were estimated using g-computation [27] and complemented by cluster-robust standard errors [28] using the R “marginal effects” library.

## 3. Results

### 3.1. Patient Characteristics

Six hundred seven patients with periampullary malignancies who underwent PD were evaluated for eligibility. Patients who were diagnosed with benign disease (N = 104) or lacked follow-up information (N = 11) or data regarding BT, blood loss volume, or Hb (N = 1) level, were excluded from the study. Of the remaining 491 patients who met the inclusion criteria and were included in the analysis, 18 (3.7%) received at least one unit of blood during intraoperative BT. Of these, 14 patients received one unit, three received three, and one received four. In none of the 18 patients who received intraoperative BT (95% CI: 0% to 15.5%) were adverse events related to intraoperative BT. The PDs were performed by four surgeons, collectively responsible for 80.9% of the procedures. The remaining 19.1% were performed by another four surgeons. There were no significant differences observed between surgeons with respect to intraoperative BT (*p* = 0.120). Additionally, when classifying periampullary tumors according to histo-anatomical origin, no significant differences were observed between groups with respect to intraoperative BT (*p* = 0.888). Patients who received intraoperative BT had higher ASA classes, either III or IV (77.8 vs. 47.6%; *p* = 0.012), lower preoperative Hb levels (106.5 vs. 127.0 g/L; *p* < 0.001), more complicated procedures such as PD type III (33. 3% vs. 13.1%; *p* = 0.042) and PD type IV procedures (5.6% vs. 1.7%; *p* = 0.042) longer operating times (485 vs. 360 min; *p* = 0.001) and more significant intraoperative blood loss (1475 vs. 500 mL; *p* = 0.001) (Table 1). In multivariable logistic regression, only intraoperative blood loss (OR per 100 mL = 1.42; 95% CI 1.27 to 1.62; *p* < 0.001) and higher ASA classes (III or IV) (OR = 3.75; 95% CI 1.05 to 17.74; *p* = 0.041) were confirmed as independent predictors for intraoperative BT.

### 3.2. Complete Case Analysis

The length of hospital stay was longer in the intraoperative BT group than in the non-intraoperative BT group (median of 22 vs. 17 days; *p* = 0.041) (Table 2). The increased rate of major postoperative complications in the intraoperative BT group was not statistically significant (50% vs. 32.1%; *p* = 0.113). The intraoperative BT and non-intraoperative BT groups presented comparable rates of pancreatic-specific morbidity. This was particularly evident in the incidence of clinically relevant (Grades B and C) POPF (16.7% vs. 20.9%; *p* = 0.706). Furthermore, comparable rates of PPH (33.3% vs. 16.5%; *p* = 0.090), bile leakage (11.1% vs. 6.6%; *p* = 0.457), and gastric anastomotic leakage (5.6% vs. 2.7%; *p* = 0.500) were observed in both groups. The postoperative BT rate exhibited no statistically significant difference between the intraoperative BT and non-intraoperative BT groups (33.3% vs. 16.5%; *p* = 0.090). No significant difference was observed in the incidence of superficial SSI (38.9% vs. 22.6%; *p* = 0.133), deep incision SSI (16.7% vs. 5.3%; *p* = 0.091) or deep organ SSI (22.2% vs. 16.7%; *p* = 0.534) between both groups. The 90-day postoperative mortality rate was comparable between the BT and No-BT groups (11.1% vs. 8.9%; *p* = 0.704).

A non-parametric partial correlation analysis was performed to elaborate further on the association between postoperative complications in terms of the CD classification with the number of intraoperative BT, intraoperative blood loss, and preoperative Hb levels. The severity of postoperative complications was correlated with intraoperative blood loss (r = 0.12. *p* = 0.002) but not with intraoperative BT (r = 0.02, *p* = 0.698), and not with preoperative Hb levels (r = 0.04, *p* = 0.376). Intraoperative BT was positively correlated with intraoperative blood loss (r = 0.27, *p* < 0.001) and negatively correlated with the preoperative Hb levels (r = −0.16, *p* < 0.001).

When adjusted for the main surgeon, study center, PD difficulty, and UICC stage, intraoperative BT was associated with major complications in the unadjusted cohort (OR= 1.95; 95% CI 1.38 to 2.42, *p* < 0.001) but not in the multivariable-adjusted ordinal mixed-effects regression analysis (OR= 0.75; 95% CI 0.37 to 1.48, *p* = 0.001) (Table 3). Major complications were associated with longer durations of surgery (per hour OR= 1.12; 95% CI: 1.04 to 1.21; *p* = 0.002), higher BMI (per kg/m^2^ OR= 1.04; 95% CI: 1.01 to 1.07; *p* = 0.008), and greater blood loss volumes (per 100 mL OR = 1.03; 95% CI: 1.00 to 1.06; *p* = 0.081). These findings were further confirmed in a mediation analysis, which proved the effect of intraoperative BT on the incidence of major complications (relative risk (RR) = 0.51; 95% CI: 0.01–0.78) and the intraoperative blood loss volume (per 100 mL RR = 2.49; 95% CI: 1.75–177.34).

### 3.3. PSM Analysis of Perioperative Outcomes

**Figure 1:**
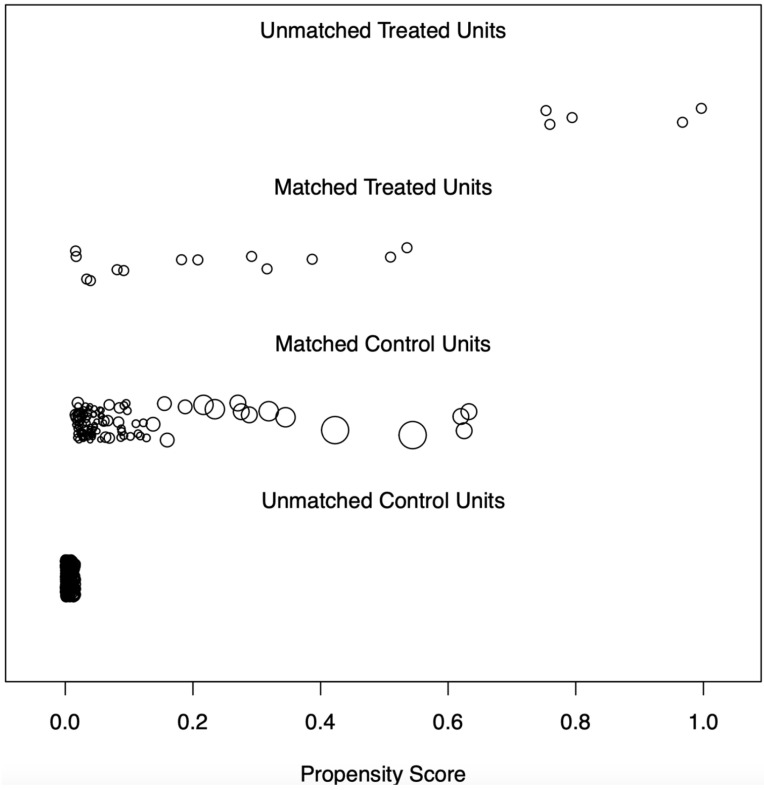
Distribution of Propensity Scores. Each circle presents one patient. The distribution of the propensity scores for patients with intraoperative blood transfusion (treatment units) versus non-intraoperative blood transfusion (control units) who could be matched is shown. The propensity scores for patients who could not matched because their characteristics could not be matched with patients from the other group are also shown. The size of the circle for patients with non-intraoperative blood transfusion (“control units”) represents the weight obtained by the propensity score matching procedure.

The intraoperative BT and non-intraoperative BT groups were highly biased, with propensity scores of 0.388 ± 0.341 and 0.023 ± 0.070, respectively (*p* < 0.001). PSM yielded a well-matched but small sample of patients in both groups (BT, N = 13; No-BT, N = 100) with similar propensity scores (0.208 ± 0.185 vs. 0.221 ± 0.200, *p* = 0.815) (Figure 1). A total of 5 and 373 patients in the intraoperative and non-intraoperative BT groups, respectively, could not be matched with patients in the other groups. These patients had to be excluded because no counterpart in the other group matched their characteristics. In the remaining cohort (N = 113) after PSM, the maximum absolute imbalance in terms of SMD was 0.074, indicating a good balance not only for the propensity score but also for all confounding variables.

After PSM, the intraoperative BT and non-intraoperative BT groups exhibited only a difference in preoperative Hb levels (106.2 vs. 124.2 g/L, *p <* 0.001). No significant differences were observed between the two groups in terms of blood loss volume (1220 vs. 1200 mL, *p* = 0.856), duration of surgery (7.5 vs. 7.5 h, *p* = 0.948), PD difficulty: standard (46.2% vs. 45.3%, *p* = 0.942), age at surgery (64.0 vs. 63.9 years, *p* = 0.984), male sex (38.5% vs. 35.3%; *p* = 0.834), BMI (25.3 vs. 25.1 kg/m^2^, *p* = 0.944) or ASA class (III or IV) (76.9% vs. 76.8%, *p* = 0.995).

No significant impact of intraoperative BT on major postoperative complications (OR = 1.05; 95% CI: 0.42 to 2.60, *p* = 0.919), clinically relevant POPF grades B and C (OR = 0.57; 95% CI: 0.14 to 2.35, *p* = 0.439), PPH (OR = 0.71, 95% CI: 0.20 to 2.62, *p* = 0.439) or postoperative 90-day mortality (OR = 0.71, 95% CI: 0.20 to 2.62, *p* = 0.439) was found after PSM (Table 4).

## 4. Discussion

The present study revealed that intraoperative BT was associated with blood loss and relatively high ASA classes, but there was no association between intraoperative BT and postoperative complications when intraoperative blood loss was adjusted for. The results of the mediation analysis indicated that an intraoperative BT might be beneficial for reducing the risk of major postoperative complications and confirmed the detrimental effect of intraoperative blood loss on the incidence of major postoperative complications. The incidence of PPH, POPF, biliary fistula, and gastric anastomotic fistula was found to be twice as high in the intraoperative BT group compared to the non-intraoperative BT group in the unmatched cohort group. However, this difference was not statistically significant. Following the application of PSM, the patients who underwent intraoperative BT presented comparable rates of pancreas-specific morbidity, particularly clinically relevant POPFs, and an incidence of SSI comparable to that in patients who did not receive an intraoperative BT. A significant association was identified between intraoperative BT and estimated intraoperative blood loss and Hb level, and between estimated intraoperative blood loss and major postoperative complications.

PD represents the sole curative treatment option for patients with periampullary malignancies. A review of existing studies revealed that a perioperative BT is needed in approximately 68% of patients who undergo PD [29,30]. Importantly, the increase in complex vascular reconstructions in PD, which are well known to require perioperative BT, has not been reported thus far. Despite studies [3,5,31,32] examining the postoperative short-term outcomes of BT in patients undergoing PD for periampullary malignancies, definitive conclusions remain elusive owing to substantial discrepancies in clinical practice as well as adherence to transfusion guidelines and the fact that perioperative BT has been employed. Owing to this information, the aim of this first partial correlation analysis and mediation analysis complemented by PSM, and weighting was to investigate the impact of intraoperative BT on postoperative short-term outcomes after PD.

The prevalence of severe postoperative complications resulting from intraoperative BT observed in the present study differs from that reported in previous studies of patients with PD. A review of the relevant literature revealed that postoperative BT is an independent risk factor for SSIs [3,33,34]. An analysis of the ACS-NSQIP data (n = 4817), comprising patients who underwent PD, indicated a graded association between perioperative BT and 30-day morbidity [31]. While the studies mentioned above have examined the impact of perioperative BT on perioperative morbidity [3,31,33,34], there is currently no research investigating the influence of intraoperative BT on the occurrence of major complications in patients with PD. The inconsistency of results across our study and previously published literature is because all the studies above compared perioperative (intraoperative and postoperative) BT with postoperative complications. It is challenging to ascertain the impact of perioperative BT on postoperative complications, mainly when patients receive a postoperative BT because of complications such as POPF or PPH. Additionally, more objective assessments must evaluate the necessity of intraoperative BT in this patient group.

Despite considerable advances in surgical techniques and increasing experience in performing PD, the risk of intraoperative blood loss remains a significant concern. Previous studies have revealed that intraoperative BT rates for this procedure range from 45% to 60% [11,35]. Importantly, researchers have not fully accounted for the increasing trend in complex vascular reconstructions in PD patients despite such patients possibly requiring a BT. Therefore, the relationships between intraoperative blood loss and the incidence of complications, as well as intraoperative BT, were examined in this study. An association was identified between the intraoperative BT rate and both the intraoperative blood loss volume and the postoperative complication rate. Therefore, intraoperative BT is required for patients with major intraoperative blood loss or low preoperative Hb levels who are undergoing PD. It can thus be concluded that it is not the intraoperative BT that causes complications but rather intraoperative blood loss or preoperative untreated anemia that is responsible for the higher rate of postoperative complications reported in the literature. Our findings align with previously published research indicating that patients who received an intraoperative BT presented with lower preoperative Hb levels [8,35], higher estimated intraoperative blood loss volumes [8,30,36,37], and a suboptimal preoperative physical condition [8,36]. Advanced disease status has also emerged as a contributing factor [37].

The finding that intraoperative BT is not an independent risk factor for postoperative complications and that intraoperative blood loss itself significantly increases the risk of postoperative complications has significant clinical implications, as it allows for a more differentiated approach to transfusion therapy. For example, it may be beneficial to consider not minimizing the amount of blood administered or performing a BT preoperatively in patients with anemia. Furthermore, transfusion of blood may be beneficial for patients who bleed during PD. Following current clinical guidelines, a BT is typically recommended only when a patient’s Hb level falls below 7–8 g/dL [38,39,40]. This guideline was also followed for our patient cohort. In this context, it is pertinent to consider whether adjusting the cutoff value for the number of blood units transfused is necessary. Based on the present study’s findings, it is impossible to provide a definitive answer to this question. A literature review revealed that several previous studies have already addressed the issue of postoperative BT cutoff values. Compared with the liberal utilization of BT, the restrictive use of BT may lead to postoperative morbidity [13,40,41,42,43]. The data from a meta-analysis revealed that nearly half of all patients who underwent pancreatic surgery for cancer received a perioperative BT [10]. The presented data and the literature previously described indicate that an evidence-based strategy based on personalized transfusion triggers should be more widely adopted to avoid the unnecessary, harmful effects of BT in patients or to avoid exposing a patient to the risks associated with non-transfusion.

The intraoperative BT rate observed in the current study was lower than that reported in previous research studies, which have documented a range of 5–57% [3,5,11,44]. The discrepancies observed can be attributed to the varying criteria used to determine the necessity for transfusions. Furthermore, it is crucial to take into account the discrepancies in the populations under investigation. For example, in some studies, the administration of BT was validated based on symptoms such as dyspnea and chest pain, which are not suitable for assessing intraoperative BT. In other studies, the transfusion threshold was set at an intraoperative haematocrit of less than 30% in the absence of angina pectoris or a history of myocardial infarction within the previous six weeks prior to surgery. At our facilities, intraoperative BT was only conducted when the Hb level was below 70 g/L or in cases of coronary artery disease when the Hb level had decreased below 80 g/L. Secondly, a preoperative screening process was conducted to identify any patients with anemia. If anemia was identified, iron replacement therapy, if feasible, was initiated to address the underlying anemia. Thirdly, our two centers are amongst the largest in Switzerland, with particular expertise in the field of pancreatic surgery.

Notably, the current study is characterized by various limitations. These limitations include the retrospective nature of the study, the analysis of a limited number of patients who receive an intraoperative BT, and the 14-year study period. For example, an understanding of the perioperative cytokine profile and the composition of peripheral blood T lymphocyte subsets could facilitate the investigation of the mechanism of immunosuppression in patients who require a perioperative BT. The relative risk associated with some variables may have changed over the 14-year study period. The retrospective study design introduces potential bias due to patient selection and uncontrolled between-group differences in baseline characteristics. Although the investigators performed PSM and mediation analysis to minimize differences in several baseline characteristics, other confounding factors may have influenced the results. Although the criteria for blood transfusion have been clearly defined, the decision to transfuse blood units was made as a result of collaboration between the surgeon and the anesthetist or, in some cases, the anesthetist alone. It is possible that differences in transfusion practice patterns between different anesthetists were not accounted for, which could have affected the results.

## 5. Conclusions

In conclusion, based on our study data intraoperative BT is not an independent risk factor for major complications in patients with periampullary malignancies following PD. This is of clinical relevance because BT in patients undergoing PD can be considered on an as needed base without the concern of worsening postoperative outcomes. 

## Figures and Tables

**Table 1:** The demographic, clinicopathological, and surgical characteristics of the entire study cohort stratified by the receipt of intraoperative blood transfusion before and after propensity score matching.

Characteristics	Unmatched Cohort, n (%)			*p* Value
	Total (n = 491)	No-BT (n = 473)	BT (n = 18)	
Baseline characteristics				
Age, years, median (IQR)	68.2 (59.3–75)	68.2 (59.3–75)	66.8 (57–72.8)	0.387 ^a^
Obesity (BMI > 30 kg/m^2^)	60 (12.2)	56 (11.8)	4 (22.2)	0.082 ^a^
Sex, Male	201 (40.9)	194 (41)	7 (38.9)	0.857 ^b^
ASA class ≥ 3	239 (48.7)	225 (47.6)	14 (77.8)	0.012 ^b^
Preoperative Hb (g/L), median (IQR)	127 (115–137)	127 (116–137)	106 (100–119)	<0.001 ^a^
Coexisting condition				
Diabetes mellitus	90 (18.4)	87 (18.4)	3 (16.7)	0.814 ^b^
Bleeding disorder	28 (5.7)	26 (5.5)	2 (11.1)	0.350 ^d^
Corticosteroid use	22 (4.5)	21 (4.4)	1 (5.6)	1.000 ^b^
History of abdominal surgery	102 (20.8)	97 (20.5)	5 (27.8)	0.460 ^d^
Smoking history	199 (40.5)	194 (41)	5 (27.8)	0.232 ^b^
Neoadjuvant chemotherapy	43 (8.8)	39 (8.2)	4 (22.2)	0.078 ^d^
Tumor localization				0.723 ^c^
Pancreas head	344 (70.1)	330 (69.8)	14 (77.8)	
Pancreas corpus	5 (1)	5 (1.1)	0 (0)	
Ampullary	66 (13.4)	64 (13.5)	2 (11.1)	
Duodenum	35 (7.1)	35 (7.4)	0 (0)	
Distal bile duct	41 (8.4)	39 (8.2)	2 (11.1)	
Surgical characteristics				
Open approach	485 (98.8)	468 (98.9)	17 (94.4)	0.219 ^b^
Extent of PD according to Mihaljevic [2]				0.042 ^c^
Type I	365 (74.3)	357 (75.5)	8 (44.4)	
Type II	49 (10)	46 (9.7)	3 (16.7)	
Type III	68 (13.8)	62 (13.1)	6 (33.3)	
Type IV	9 (1.8)	8 (1.7)	1 (5.6)	
Main Surgeon				0.120 ^b^
Surgeon I	120 (24.4%)	117 (24.7%)	3 (16.7%)	
Surgeon II	117 (23.8%)	109 (23.0%)	8 (44.4%)	
Surgeon III	91 (18.5%)	90 (19.0%)	1 (5.6%)	
Surgeon IV	69 (14.1%)	68 (14.4%)	1 (5.6%)	
Other	94 (19.1%)	89 (18.8%)	5 (27.8%)	
Duration of Surgery, min., median (IQR)	360 (296–465.5)	360 (294–458)	485 (371.2–525.5)	0.001 ^a^
Estimated blood loss (mL), median (IQR)	500 (300–700)	500 (300–700)	1475 (1050–1875)	0.001 ^a^
Pathological characteristics				
Histological subclassification				0.888 ^c^
Pancreatic ductal adenocarcinoma	303 (61.7%)	291 (61.5%)	12 (66.7%)	
Non-pancreatic periampullary carcinoma	144 (29.3%)	139 (29.4%)	5 (27.8%)	
Neuroendocrine tumor	44 (9.0%)	43 (9.1%)	1 (5.6%)	
UICC stage				0.599 ^c^
I	83 (16.9)	79 (16.7)	4 (22.2)	
II	279 (56.8)	268 (56.7)	11 (61.1)	
III/IV	129 (26.3)	126 (26.6)	3 (16.7)	

Abbreviations: ASA: American society of anesthesiologists; BMI: body mass index; BT: allogeneic blood transfusion; Hb: hemoglobin; IQR: interquartile range; PD: pancreatoduodenectomy; UICC: Union internationale contre le cancer. Notes: ^a^ Mann-Whitney U test; ^b^ Chi-square test; ^c^ Chi-square test, MC simulated; ^d^ Mid-p test.

**Table 2:** Incidence of postoperative complications of PD in patients who received and did not receive an intraoperative blood transfusion.

Postoperative Complications	Unmatched Cohort, n (%)			*p* Value
	Total (n = 491)	No-BT (n = 473)	BT (n = 18)	
Complications according Clavien-Dindo				0.236 ^c^
Grade 0	7 (1.4)	6 (1.3)	1 (5.6)	
Grade 1	120 (24.4)	117 (24.7)	3 (16.7)	
Grade 2	203 (41.3)	198 (41.9)	5 (27.8)	
Grade 3a	47 (9.6)	45 (9.5)	2 (11.1)	
Grade 3b	29 (5.9)	28 (5.9)	1 (5.6)	
Grade 4a	36 (7.3)	33 (7)	3 (16.7)	
Grade 4b	14 (2.9)	12 (2.5)	2 (11.1)	
Grade 5	35 (7.1)	34 (7.2)	1 (5.6)	
Clavien-Dindo grade ≥ 3a	161 (32.8)	152 (32.1)	9 (50)	0.113 ^b^
Reoperation	92 (18.7)	87 (18.4)	5 (27.8)	0.334 ^a^
In hospital mortality rates	35 (7.1)	34 (7.2)	1 (5.6)	1.000 ^a^
Pancreatic-specific complications				
Postoperative pancreatic fistula				0.735 ^c^
Grade A	34 (6.9)	32 (6.8)	2 (11.1)	
Grade B	63 (12.8)	62 (13.1)	1 (5.6)	
Grade C	39 (7.9)	37 (7.8)	2 (11.1)	
Postpancreatectomy hemorrhage				
Grade A–C	88 (17.9)	82 (17.3)	6 (33.3)	0.111 ^a^
Delayed gastric emptying				
Grade A–C	173 (35.2)	166 (35.1)	7 (38.9)	0.741 ^b^
Bile leakage	33 (6.7)	31 (6.6)	2 (11.1)	0.457 ^a^
Gastroenteric anastomotic leakage	14 (2.9)	13 (2.7)	1 (5.6)	0.500 ^a^
Surgical site infection (SSI)				
Superficial	114 (23.2)	107 (22.6)	7 (38.9)	0.133 ^a^
Deep incisional	28 (5.7)	25 (5.3)	3 (16.7)	0.091 ^a^
Deep organ/space	83 (16.9)	79 (16.7)	4 (22.2)	0.534 ^a^
Postoperative BT	84 (17.1)	78 (16.5)	6 (33.3)	0.090 ^a^
Postoperative complications summary				
Length of ICU stay, hours, median (IQR)	24.0 (18.0–48.0)	24.0 (18.0–48.0)	45.5 (20.8–93.8)	0.155 ^d^
Length of Hospital stay, days, median (IQR)	17.0 (13.0–26.0)	17.0 (13.0–26.0)	22.0 (18.5–47.0)	0.041 ^d^
Postoperative mortality rates				
30–days	22 (4.5)	21 (4.4)	1 (5.6)	0.759 ^a^
90–days	44 (9)	42 (8.9)	2 (11.1)	0.704 ^a^

Abbreviations: BT: allogeneic blood transfusion; ICU: intensive care unit; IQR: interquartile range. Notes: ^a^ Mid-p test; ^b^ Chi-square test; ^c^ Chi-square test, MC simulated; ^d^ Mann-Whitney U test.

**Table 3:** Mixed effects ordinal regression analysis of the severity of complications graded using the Clavien–Dindo classification system.

Levels	Univariable Analysis OR (95% CI) ^a^	*p* Value	Multivariable Analysis OR (95% CI) ^b^	*p* Value
Intraoperative BT				
No	Reference		Reference	
Yes	2.01 (1.14–2.88)	0.020	0.77 (0.50–1.25)	0.210
Estimated blood loss per 100 mL				
Continuous	1.04 (1.01–1.05)	0.005	1.02 (1.00–1.04)	0.020
Duration of surgery				
Continuous	1.16 (1.12–1.24)	0.005	1.08 (1.02–1.16)	0.030
Extent of PD according to Mihaljevic et al. [2]				
Type I	Reference		Reference	
Type II	1.18 (0.82–1.55)	0.300	1.07 (0.84–1.43)	0.660
Type III	1.49 (1.10–1.92)	0.010	1.38 (1.04–1.79)	0.005
Type IV	1.86 (0.89–4.07)	0.080	1.61 (0.92–3.07)	0.140
Age at surgery				
Continuous	1.01 (1.00–1.02)	0.060	1.01 (1.00–1.02)	0.030
Sex				
Male	Reference		Reference	
Female	1.29 (1.06–1.63)	0.032	1.18 (0.98–1.37)	0.130
BMI				
Continuous	1.03 (1.02–1.05)	0.005	1.03 (1.01–1.05)	0.005
ASA class				
I/II	Reference		Reference	
III/IV	1.20 (1.04–1.42)	0.020	1.12 (0.92–1.38)	0.280

Abbreviations: ASA: American Society of Anesthesiologists; BMI: body mass index; BT: allogeneic blood transfusion; PD: pancreatoduodenectomy; OR: odds ratios with 95% confidence intervals (Wald type). Notes: *p* values are derived using the likelihood ratio test. A higher OR indicates a worse outcome. ^a^ Univariate logistic regression analysis. ^b^ Multivariate logistic regression analysis.

**Table 4:** Incidence of postoperative complications of PD in patients who received and did not receive an intraoperative blood transfusion after PSM (N = 113).

Postoperative Complications	Propensity Score Matched Cohort			*p* Value
	No-BT, n (%)	BT, n (%)	OR (95% CI)	
Major complication according the Clavien-Dindo classification				
CD Grade ≥ 3a	36.7 (36.7)	5 (38.5)	1.048 (0.423–2.597)	0.919
Reoperation	27.3 (27.3)	2 (15.4)	0.564 (0.130–2.446)	0.444
Postoperative pancreatic fistula				
Grade A–C	35.4 (35.4)	3 (23.1)	0.651 (0.196–2.161)	0.483
Postoperative pancreatic fistula				
Grade A	8.6 (8.6)	1 (7.7)	0.894 (0.105–7.583)	0.918
Grade B	8.1 (8.1)	1 (7.7)	0.949 (0.126–7.168)	0.960
Grade C	18.7 (18.7)	1 (7.7)	0.411 (0.045–3.766)	0.431
Clinically relevant postoperative pancreatic fistula				
Grades B and C	26.8 (26.8)	2 (15.4)	0.573 (0.140–2.351)	0.440
Postpancreatectomy hemorrhage				
Grade A–C	32.3 (32.3)	3 (23.1)	0.714 (0.195–2.623)	0.612
Bile leakage	10.9 (10.9)	1 (7.7)	0.706 (0.073–6.798)	0.763
Delayed gastric emptying				
Grade A–C	28 (28)	4 (30.8)	1.100 (0.482–2.510)	0.821
Gastroenteric anastomotic leakage	4.7 (4.7)	0 (0)	– ^a^	– ^a^
Surgical site infection (SSI)				
Superficial	33.7 (33.7)	4 (30.8)	0.913 (0.325–2.565)	0.862
Deep incisional	10 (10)	1 (7.7)	0.770 (0.064–9.321)	0.837
Deep organ/Space	20.7 (20.7)	2 (15.4)	0.742 (0.145–3.789)	0.720
Postoperative mortality rates				
30–days	16.2 (16.2)	1 (7.7)	0.475 (0.050–4.508)	0.517
90–days	21.9 (21.9)	2 (15.4)	0.702 (0.219–2.252)	0.552

Abbreviations: BT: allogeneic blood transfusion; CD: Clavien-Dindo; OR: odds ratios with 95% confidence intervals (Wald type). Notes: *p* values are derived using the likelihood ratio test. OR and *p* values for the effect of blood transfusion were estimated via logistic regression with B-computing. ^a^ No valid estimates for gastrointestinal anastomotic leakage.

## Data Availability

The original contributions presented in the study are included in the article, further inquiries can be directed to the corresponding author.
